# Supplementary material for: Controlling the selectivity of the hydrogenolysis of polyamides catalysed by ceria-supported metal nanoparticles
Source: Nat Commun. 2023 Oct 16;14:6524. doi: 10.1038/s41467-023-42246-x (PMC10579319; doi:10.1038/s41467-023-42246-x)
Supplement: Supplementary file 1 — Supplementary Information [file 41467_2023_42246_MOESM1_ESM.pdf]

Supplementary Information

Title

Controlling the selectivity of the hydrogenolysis of polyamides catalysed by ceria-supported metal nanoparticles

Authors

XinBang Wu[1], Wei-Tse Lee[1], Roland C. Turnell-Ritson[1], Pauline C. L. Delannoi[1], Kun-Han Lin[2]*, Paul J. Dyson[1]*

Affiliations

[1]Institute of Chemical Sciences and Engineering, Swiss Federal Institute of Technology Lausanne (EPFL), Lausanne, Switzerland

[2]Department of Chemical Engineering, National Tsing Hua University (NTHU), Hsinchu, Taiwan

*Corresponding authors: Paul J. Dyson (paul.dyson@epfl.ch)

Kun-Han Lin (kunhan.lin@mx.nthu.edu.tw)

Table of Contents

Supplementary Tables

Supplementary Table 1 | Current routes for the chemical recycling of polyamides 3

Supplementary Table 2 | XPS quantification of surface metal species 6

Supplementary Table 3 | ICP-MS analysis of metal loading for M/CeO_2 7

Supplementary Table 4 | Product distribution from the conversion of N-hexylhexanamide.13

Supplementary Table 5 | Effects of temperature and reaction time, Ru/CeO_2 19

Supplementary Table 6 | Effects of temperature and reaction time, Pt/CeO_2 20

Supplementary Table 7 | Yields of non-alkane products from Supplementary Table 6...... 21

Supplementary Table 8 | C−C and C−H bond cleavage pathways for scenario I and II..... 23

Supplementary Table 9 | Yields obtained from the hydrogenolysis of PA materials 26

Supplementary Table 10 | Additional alkane products and yields with Pt/CeO_2................ 27

Supplementary Table 11 | Conversion of PA-6 at different reaction times........................ 28

Supplementary Table 12 | Recycling experiments of the Ru/CeO_2 catalyst....................... 30

Supplementary Table 13 | Recycling experiments of the Pt/CeO_2 catalyst....................... 31

Supplementary Figures

Supplementary Fig. 1 | PXRD spectra of the M/CeO_2 catalysts.. 4

Supplementary Fig. 2 | XPS analysis of the M/CeO_2 catalysts.. 5

Supplementary Fig. 3 | STEM-EDX spectra of M/CeO_2... 8

Supplementary Fig. 4 | ^{1}H NMR spectrum of N-hexylhexanamide..................................... 9

Supplementary Fig. 5 | GC-FID chromatogram showing alkane retention times................ 10

Supplementary Fig. 6 | Product characterization, Ru/CeO_2.. 11

Supplementary Fig. 7 | Product characterization, Pt/CeO_2. 12

Supplementary Fig. 8 | Yield of non-alkane products from Supplementary Table 4........... 14

Supplementary Fig. 9 | Ammonia quantification 15

Supplementary Fig. 10 | Characterization of carbon-supported catalysts 16

Supplementary Fig. 11 | Comparison of Ru-based catalysts... 17

Supplementary Fig. 12 | Effects of hydrogen pressure on hydrogenolysis 18

Supplementary Fig. 13 | | DFT computations for hydrogenolysis on Pt(111).................... 22

Supplementary Fig. 14 | Chemical structures of the polymers studied 24

Supplementary Fig. 15 | Digital images of the polymer samples studied........................... 25

Supplementary Fig. 16 | Conversion of a PA-66 fishing net 29

Supplementary Table. 1 | Current routes for the chemical recycling of polyamides.

Process	Conditions	Substrate		Product (yield)	Ref.
		Pure PA	PA-composite/ blend		
Pyrolysis (Heterogenous)	410°C Calcined scallop shells High-purity He gas flow	PA-6	–	Caprolactam (66%)	1
Hydrothermal (Homogenous)	345°C 90 bar water	PA-6	–	Caprolactam (89%)	2
Solvolysis (Homogenous)	270°C Glycolic acid MeOH	PA-66	–	Adipic acid (75%)	3
Pyrolysis (Heterogenous)	360°C KOH/α-Al_2O_3	PA-6	–	Caprolactam (85%)	4
Solvolysis (Homogenous)	300°C Ionic acid DMAP	PA-6/ PA-12	–	Caprolactam (86%)/ Laurolactam (7%)	5
Hydrogenation (Homogenous)	200°C Ru pincer complex and KO^tBu THF 100 bar H_2	PA-66	–	Diamine (78%) Diol (62%)	6
Hydrogenative ammonolysis (Heterogenous)	200°C Nb_2O_5 $RuWO_x/MgAl_2O_4$ CPME 30 bar H_2 6 bar NH_3	PA-1010/ PA-11/ PA-12	30% carbon	Primary amines (62%) Secondary amines (36%) Diamines (43%)	7
Hydrogenolysis (Heterogeneous)	325°C Ru/CeO_2 or Pt/CeO_2 50 bar H_2	Multiple PAs	30% carbon 30% glass	Methane and ammonia (>99%), or higher-order hydrocarbons and ammonia (93%)	This work

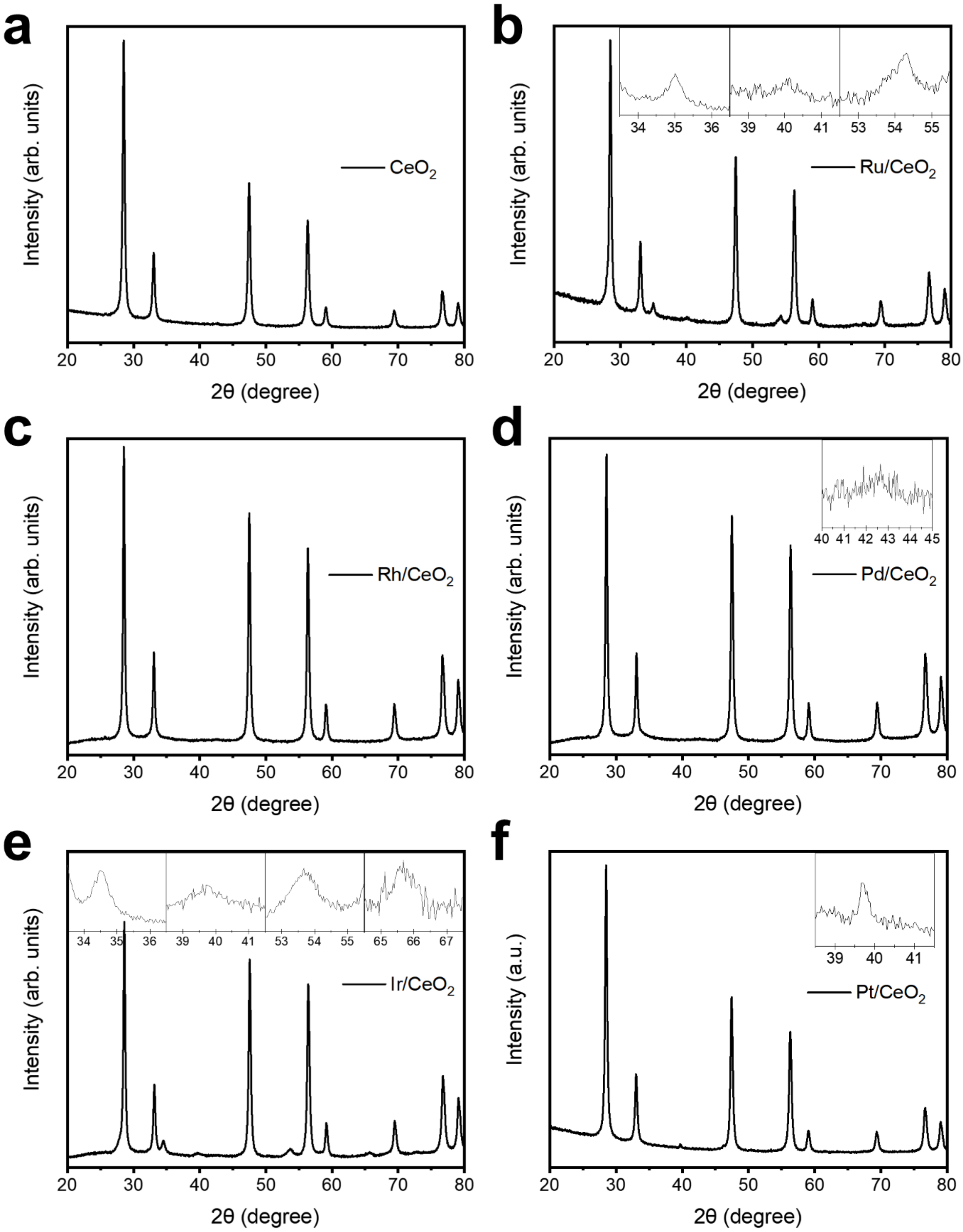

Supplementary Fig. 1 | PXRD spectra of a) CeO_2, **b)** Ru/CeO_2, inset showing RuO_2 peaks at 2θ = 35.0° (101), 40.1° (200) and 54.3° (211), **c)** Rh/CeO_2, with no visible Rh species present, **d)** Pd/CeO_2, inset showing PdO peaks at 2θ = 42.6° (110), **e)** Ir/CeO_2, inset showing IrO_2 peaks at 2θ = 34.5° (101), 39.7° (200), 53.7° (211) and 65.6 (112) and **f)** Pt/CeO_2, inset showing PtO peaks at 2θ = 39.7° (111).

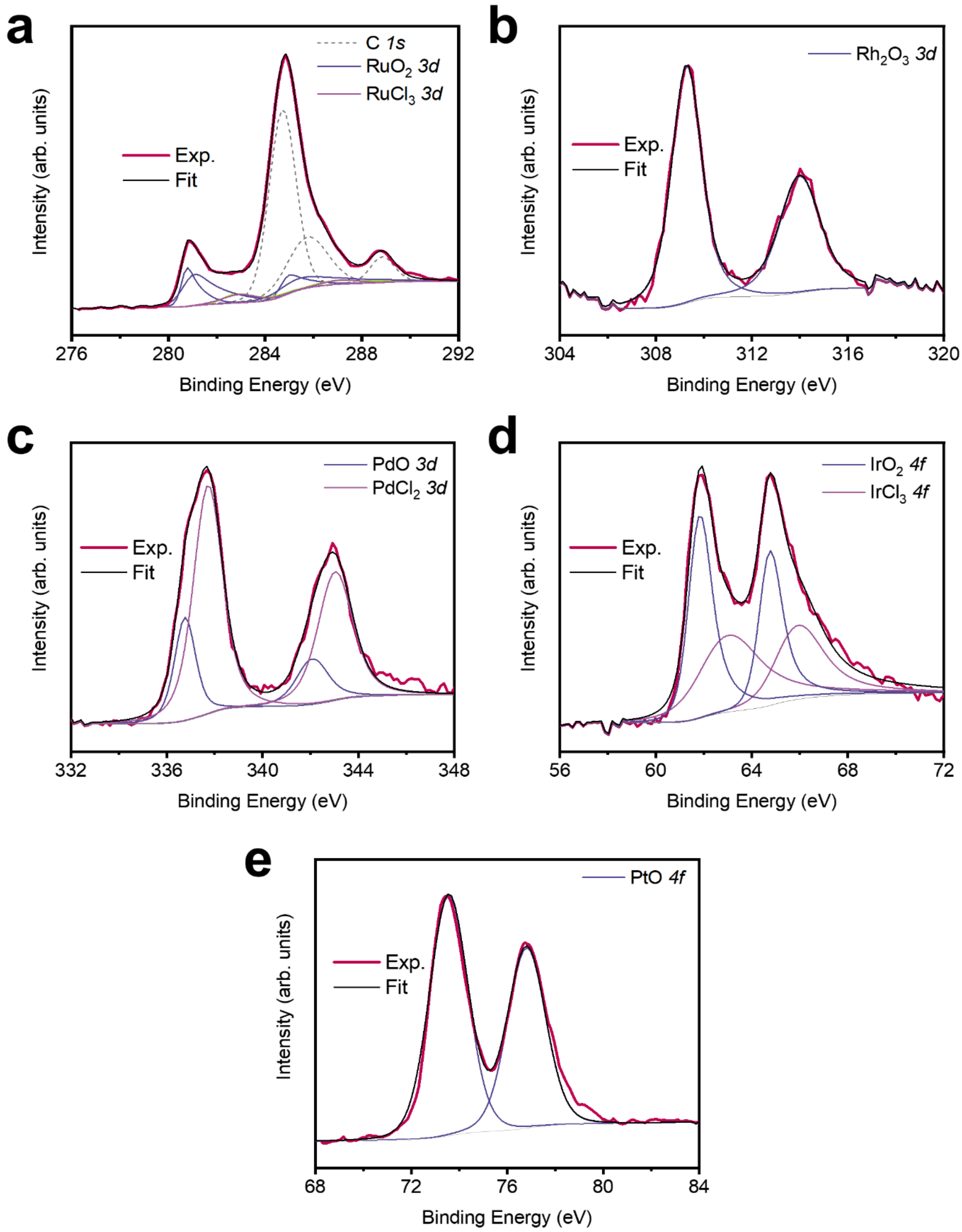

Supplementary Fig. 2 | XPS analysis of the M/CeO_2 catalysts. The main surface metal species of **a)** RuO_2 (*3d*) was detected at 280.7, 281.1, 285 and 285.4 eV, overlapping with ambient C (*1s*) peak (shown in blue), **b)** Rh_2O_3 (*3d*) was detected at 309.4 and 314 eV, **c)** PdO (*3d*) was detected at 336.8 and 342 eV, **d)** IrO_2 (*4f*) was detected at 61.9 and 64.8 eV and **e)** PtO (*4f*) was detected at 73.5 and 76.7 eV. Red corresponds to the experimental spectra and black are the theoretical fitting.

Supplementary Table. 2 | XPS quantification of surface metal species on the M/CeO_2 catalysts.

M/CeO_2 catalyst	Metal oxide (%)	Metal chloride (%)
Ru/CeO_2	89.2	10.8
Rh/CeO_2	100	−
Pd/CeO_2	24.6	75.4
Ir/CeO_2	50.6	49.4
Pt/CeO_2	100	−

Supplementary Table. 3 | ICP-MS analysis of metal loading for the M/CeO_2 catalysts.

M/CeO_2 catalyst	wt. loading of M (%)
Ru/CeO_2	4.692
Rh/CeO_2	3.650
Pd/CeO_2	3.823
Ir/CeO_2	4.325
Pt/CeO_2	5.232

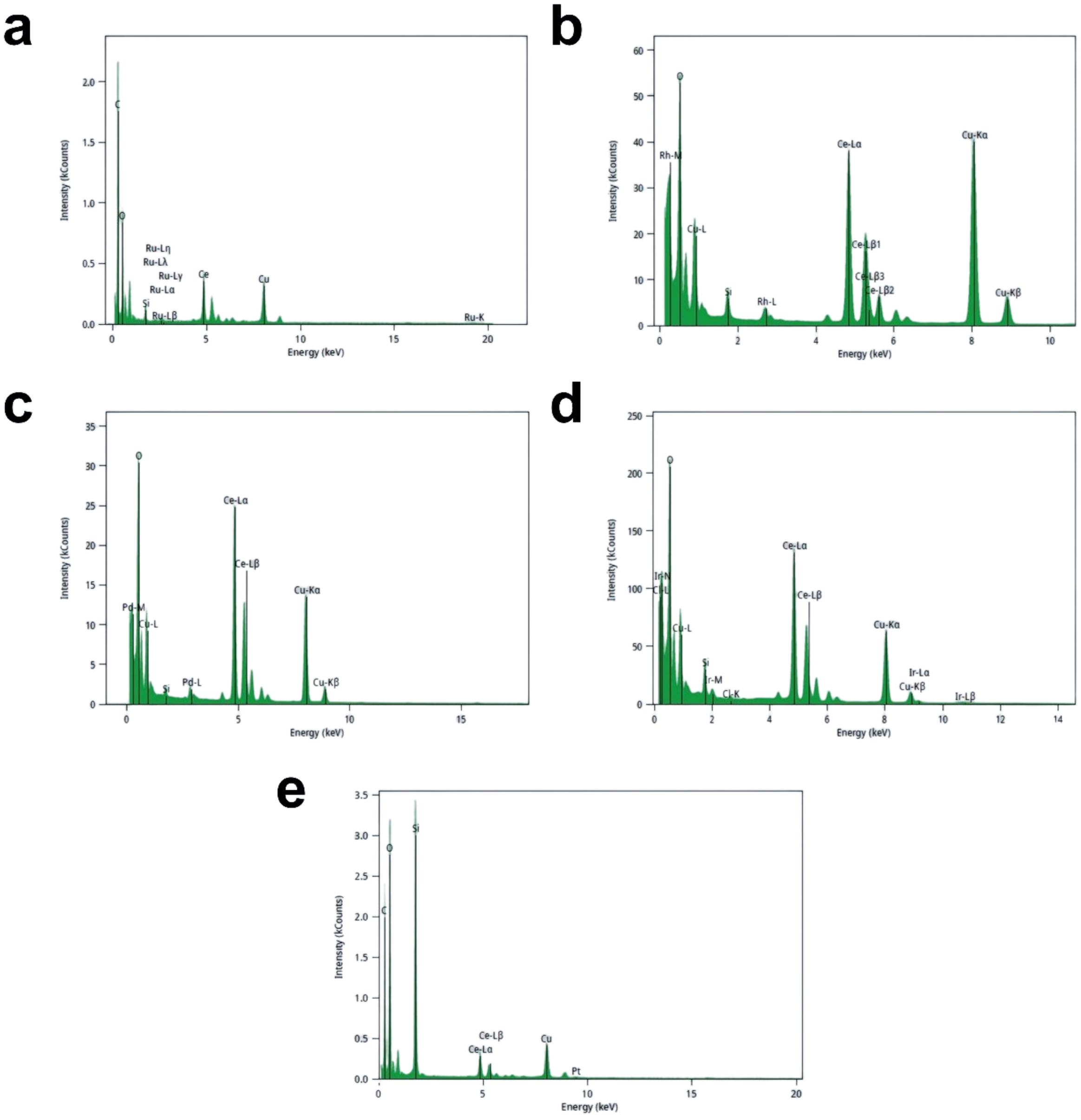

Supplementary Fig. 3 | STEM-EDX spectra of a) Ru/CeO_2, **b)** Rh/CeO_2, **c)** Pd/CeO_2 and **d)** Ir/CeO_2, and **e)** Pt/CeO_2.

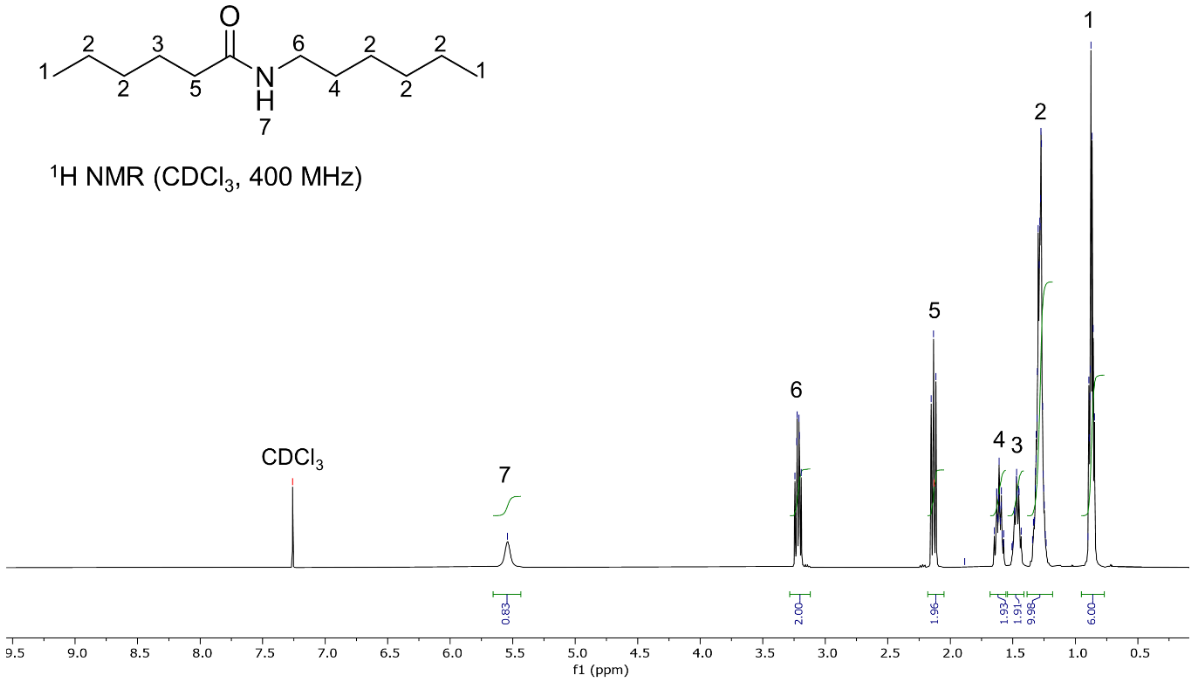

Supplementary Fig. 4 | ^{1}H NMR spectrum of N-hexylhexanamide. ^{1}H NMR (400 MHz, $CDCl_3$) δ 5.54 (s, 1H), 3.24−3.19 (q, 2H), 2.15−2.11 (t, 2H), 1.65−1.57 (m, 2H), 1.50−1.43 (m, 2H), 1.31−1.25 (m, 10H), 0.89−0.85 (m, 6H).

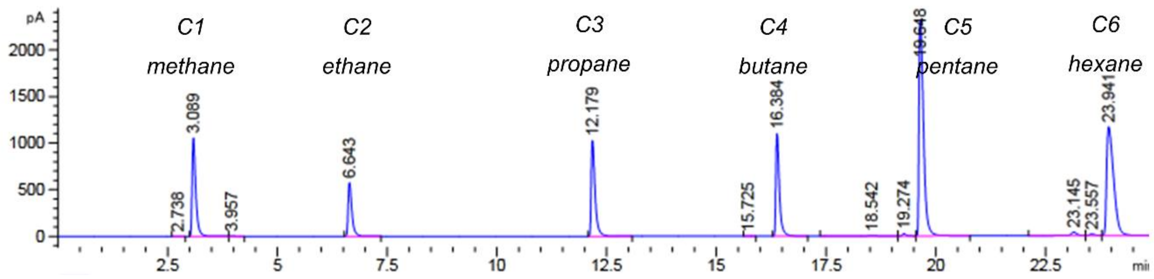

Supplementary Fig. 5 | GC-FID chromatogram showing retention time of methane to hexane. In this study, all alkane products present in the gaseous phase were characterized using GC-FID, and identified based on their retention times compared to pure C1-C6 alkanes, as shown. Product analyses for the reactions performed with Ru/CeO_2 and Pt/CeO_2 are provided as examples, as shown in Supplementary Figs. 6 and 7, respectively.

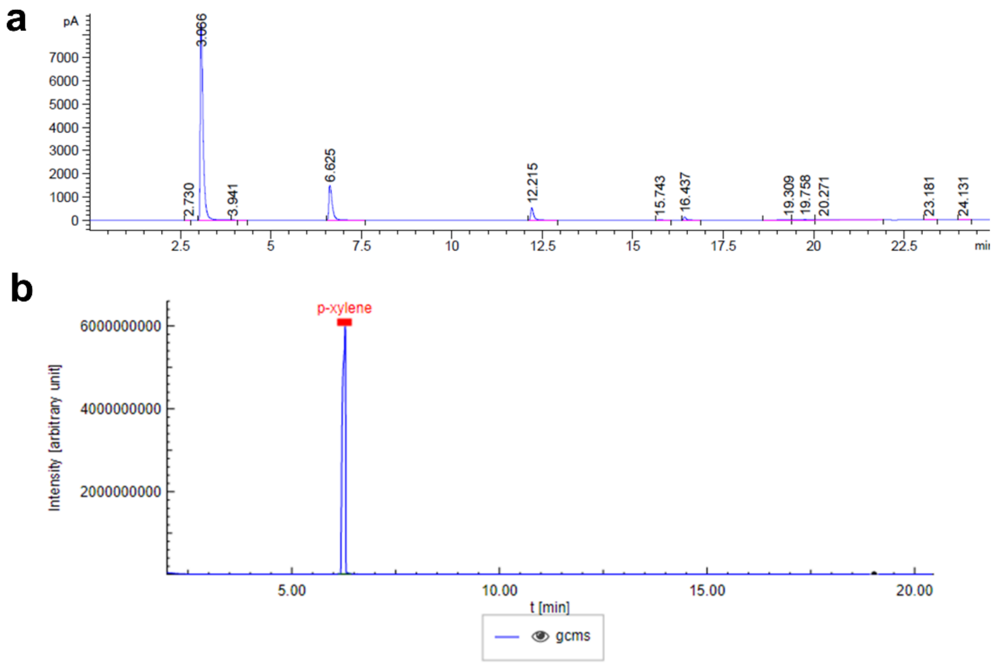

Supplementary Fig. 6 | Product characterization from the hydrogenolysis of N-hexylhexanamide with the Ru/CeO_2 catalyst. a) GC-FID spectrum of the gaseous products obtained. **b)** GC-FID spectrum of the liquid phase obtained from the reaction workup, which confirms complete conversion of the initial N-hexylhexanamide into gaseous compounds, as evidenced by the absence of any peaks other than the internal standard *p*-xylene. Reaction conditions: N-hexylhexanamide (250 mg), catalyst (26.6 mg, 0.5 wt% of Ru) and H_2 (50 bar) at 325 °C.

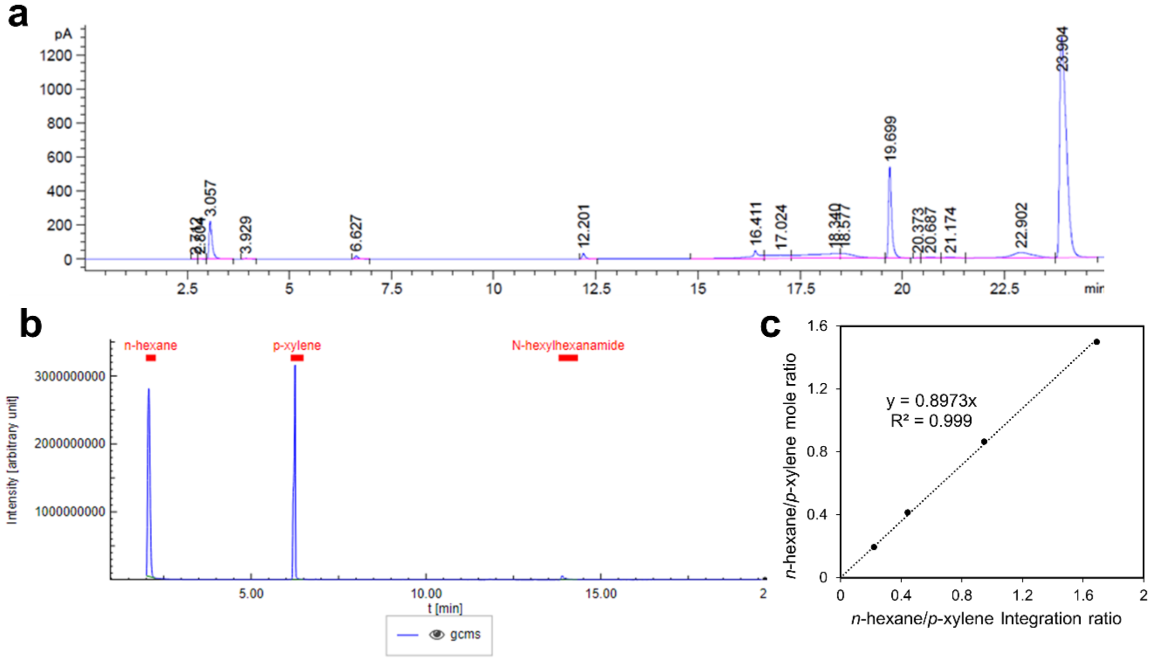

Supplementary Fig. 7 | Product characterization from the hydrogenolysis of N-hexylhexanamide with the Pt/CeO_2 catalyst. a) GC-FID spectrum of the gaseous products obtained. **b)** GC-FID spectrum of the liquid phase obtained from the reaction workup, using *p*-xylene as the internal standard. **c)** Calibration curve for measuring the amount of *n*-hexane in the liquid product was constructed using four standard solutions with different *p*-xylene/*n*-hexane ratios. The *x*-axis represents the FID integration area of *p*-xylene/*n*-hexane and the *y*-axis represents the corresponding real mass ratio of *p*-xylene/*n*-hexane. Reaction conditions: N-hexylhexanamide (250 mg), catalyst (23.8 mg, 0.5 wt% of Pt) and H_2 (50 bar) at 325 °C.

Supplementary Table 4 | Product distribution from the conversion of N-hexylhexanamide.

entry	catalyst	reaction time (h)	conv. (%)	CH_4 (%)	C_2H_6 (%)	C_3H_8 (%)	C_4H_{10} (%)	C_5H_{12} (%)	C_6H_{14} (%)	1. (%)	2. (%)	3. (%)	4. (%)	5. (%)	6. (%)
1	Ru/CeO_2	5	100	76.0 (1.5)	21.1 (2.6)	2.6 (1.0)	0.4 (0.3)	–	–	–	–	–	–	–	–
2	Rh/CeO_2	5	99.8	7.0 (0.7)	3.4 (0.7)	6.3 (1.3)	6.1 (1.3)	25 (5)	52 (3)	–	–	–	–	–	–
3	Pd/CeO_2	5	27 (8)	0.4 (0.2)	–	–	–	2.9 (1.5)	4.4 (1.9)	1.5 (0.4)	9 (2)	2.7 (0.4)	5.7 (1.5)	–	–
4	Ir/CeO_2	5	76 (10)	2.5 (0.3)	0.43 (0.02)	1.2 (0.5)	1.28 (0.16)	7.0 (0.9)	57 (10)	1.2 (0.4)	4.3 (0.7)	0.60 (0.17)	0.56 (0.19)	–	–
5	Pt/CeO_2	5	99.3 (0.3)	1.6 (0.6)	0.2 (0.06)	0.3 (0.1)	1.2 (0.6)	13.7 (1.2)	81.7 (1.4)	–	0.7 (0.3)	–	–	–	–
6	–	5	5.2	–	–	–	–	–	–	–	0.7	–	1.6	0.9	2
7	CeO_2	5	24.4	0.1	–	0.2	0.5	0.9	2.2	0.4	9	5.7	4.5	0.5	0.4
8	C	5	15.6	–	–	–	–	0.2	1.3	0.8	2.7	1.1	4	0.9	4.6
9	Ru/C	5	100	11.7 (0.8)	1.4 (0.3)	1.9 (0.3)	3.3 (0.5)	62.0 (0.7)	19.8 (1.3)	–	–	–	–	–	–
10	Ru/C	24	100	39 (5)	31 (7)	16 (5)	9 (5)	5 (3)	0.5 (0.3)	–	–	–	–	–	–
11	Pt/C	5	61 (3)	0.59 (0.03)	0.10 (0.01)	0.10 (0.01)	0.8 (0.5)	8.9 (0.3)	36 (5)	2.0 (0.5)	9.3 (0.3)	1.7 (0.4)	1.7 (0.3)	–	–
12	Pt/C	24	100	1.4 (0.3)	0.14 (0.04)	0.21 (0.05)	1.0 (0.6)	17 (5)	80 (4)	–	–	–	–	–	–

Reaction conditions: N-hexylhexanamide (250 mg), catalyst (25 mg, 0.5 wt% of metal) and H_2 (50 bar) at 325 °C. All reactions (except entries 7 and 8) were repeated three times and the reported values represent the mean percentage yield with the corresponding standard error. Chemical structures for non-alkane products **1-6** are shown in Supplementary Fig 8.

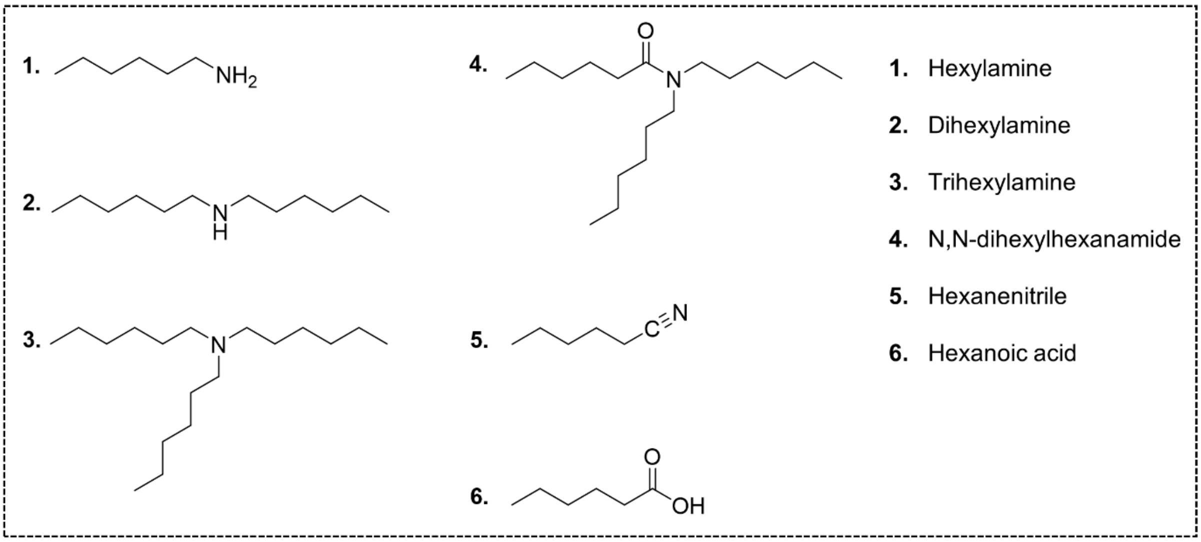

Supplementary Fig. 8 | Chemical structures for non-alkane products 1-6.

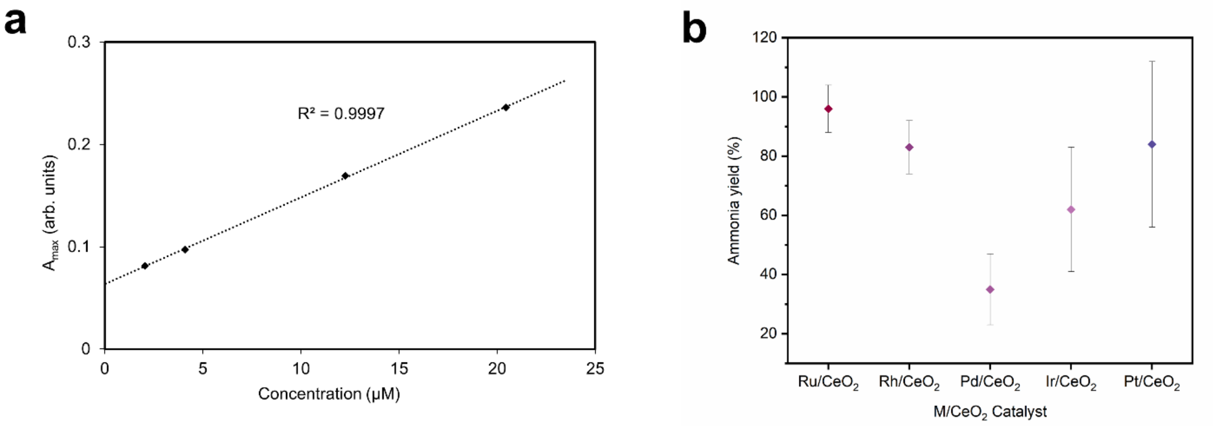

Supplementary Fig. 9 | a) Ammonia calibration curve prepared from four ammonia standards. b) Ammonia yields from the conversion of N-hexylhexanamide with the M/CeO$_2$ catalysts. The ideal gas equation, PV = nRT, was used to estimate the total volume of gas generated at the end of the reaction under ambient pressure. The amount of ammonia present in the reaction mixture was determined by analysing a small fraction (5 mL) of the gas and then scaling it up using the following equation:

$$Total\ n(NH_3) = ([NH_3] \times 10\ mL) \times \frac{overall\ V\ at\ 1\ atm}{5\ mL}$$

The presence of trace amounts of volatile amine species may contribute to an overestimation of the quantitative ammonia yields for the Ru/CeO$_2$ and Pt/CeO$_2$ catalysts.

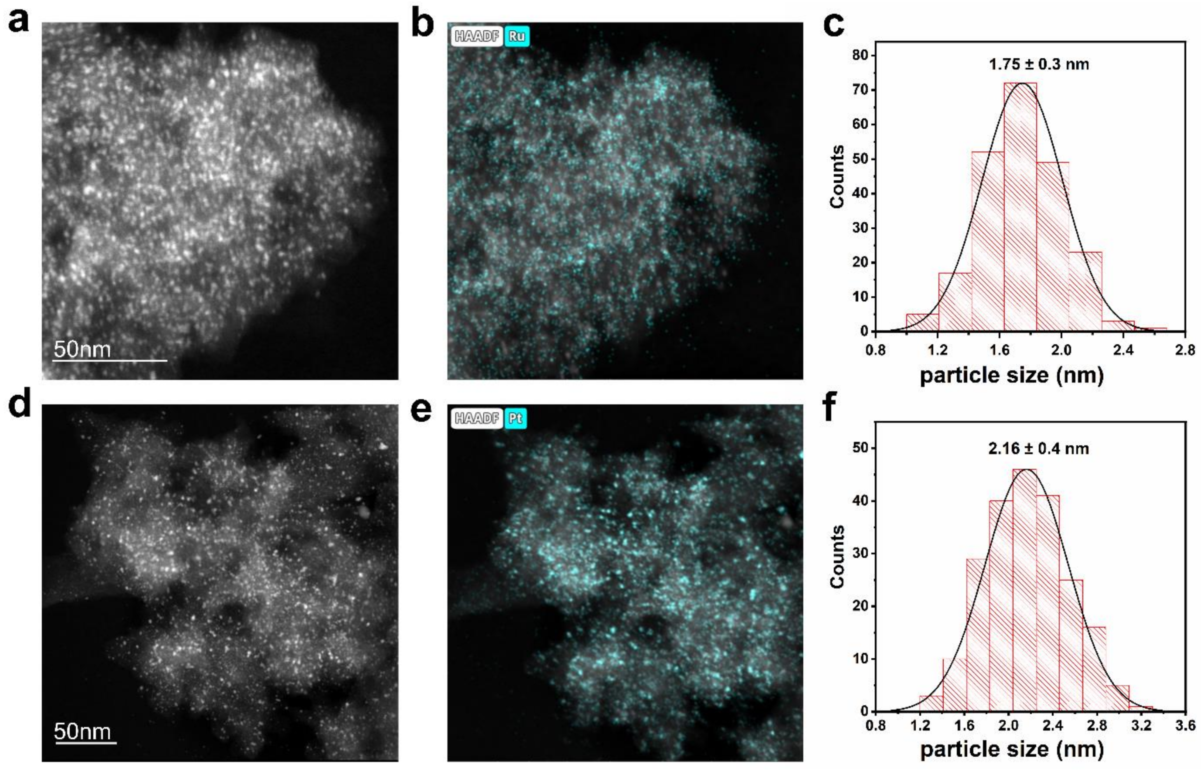

Supplementary Fig. 10 | Characterization of commercial carbon-supported catalysts. a) STEM-HAADF image of 5% Ru/C, **b)** STEM-EDX image of 5% Ru/C, **c)** nanoparticle size distribution of 5% Ru/C, **d)** STEM-HAADF image of 5% Pt/C, **e)** STEM-EDX image of 5% Pt/C and **f)** nanoparticle size distribution of 5% Pt/C.

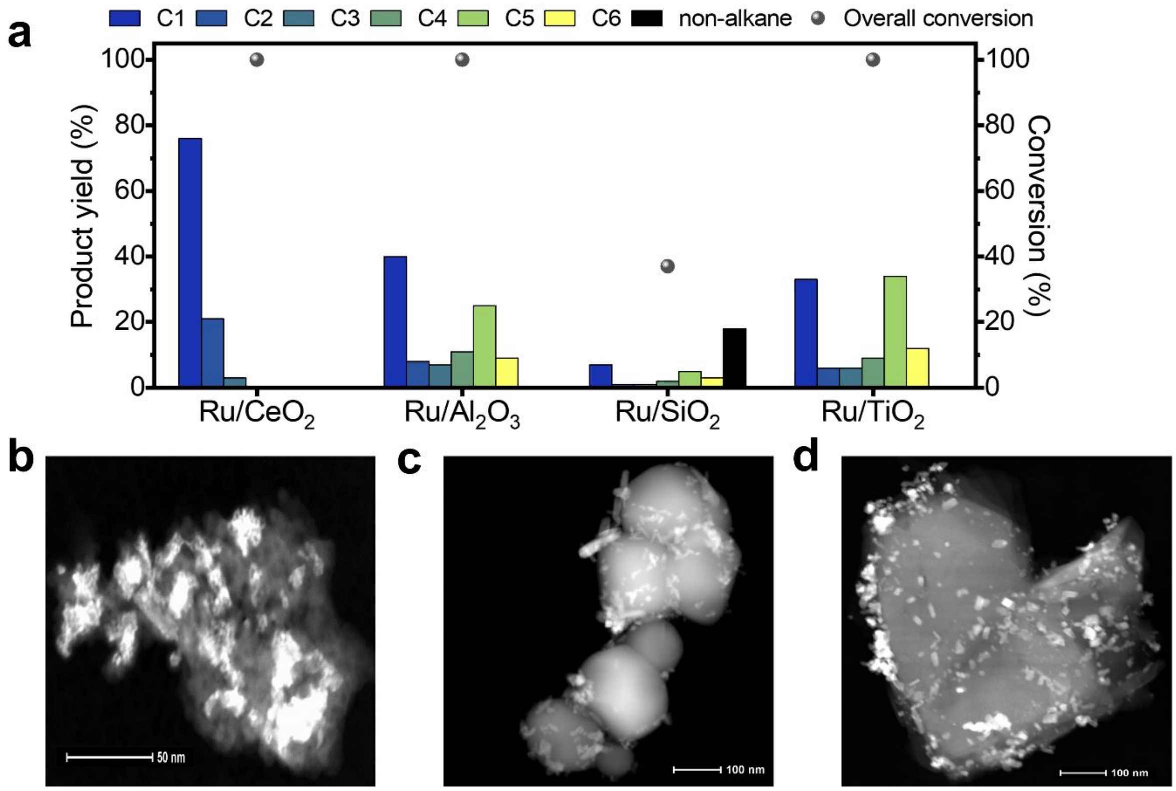

Supplementary Fig. 11 | Comparison of Ru-based catalysts with different oxide supports. a) Conversion of N-hexylhexanamide with Ru-based catalysts. **b-d)** STEM-HAADF images of b) Ru/Al_2O_3, c) Ru/ SiO_2 and d) Ru/TiO_2. The synthesis of these Ru-based catalysts followed the same procedure as that for Ru/CeO_2. Reaction conditions: N-hexylhexanamide (250 mg), catalyst (25 mg, 0.5 wt.% of metal) and H_2 (50 bar) at 325 °C.

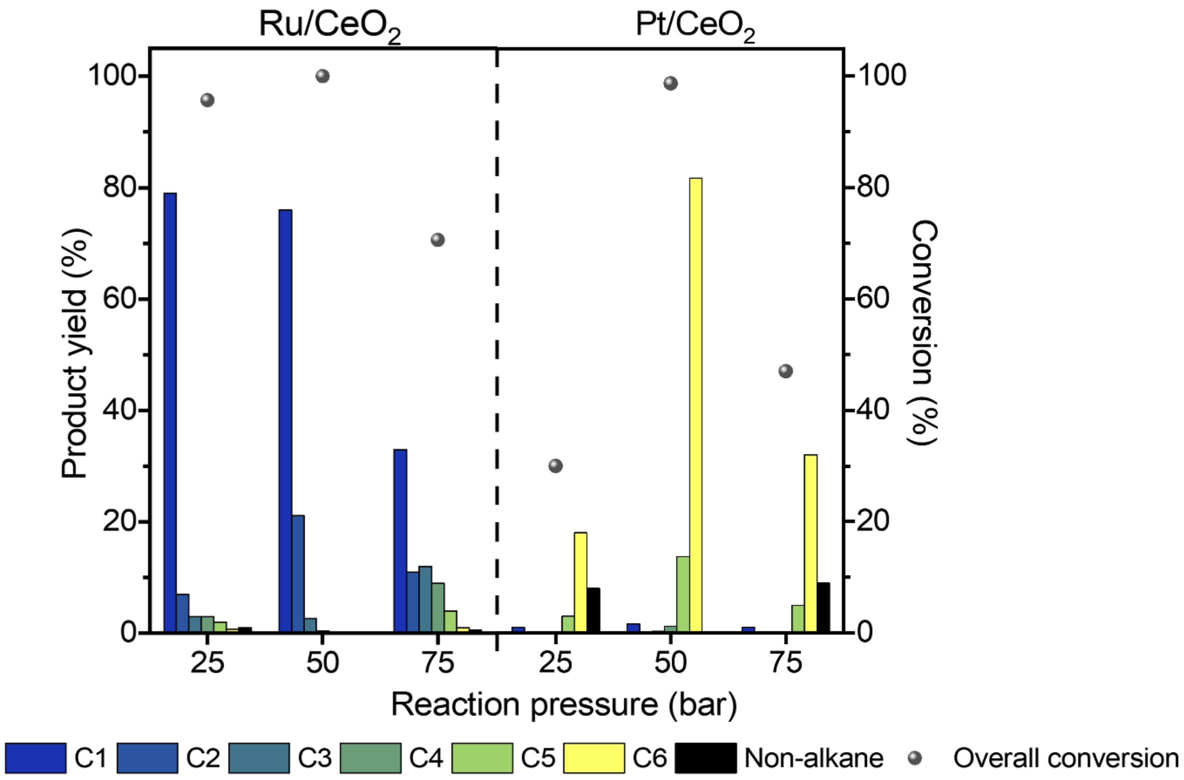

Supplementary Fig. 12 | Effects of hydrogen pressure on the hydrogenolysis of N-hexylhexanamide. Reaction conditions: N-hexylhexanamide (250 mg), catalyst (25 mg, 0.5 wt.% of metal) at 325°C under different hydrogen pressures.

Supplementary Table 5 | Effect of temperature and reaction time on the product distribution of the hydrogenolysis of N-hexylhexanamide catalysed by Ru/CeO_2.

entry	T (°C)	time (h)	conv. (%)	CH_4 (%)	C_2H_6 (%)	C_3H_8 (%)	C_4H_{10} (%)	C_5H_{12} (%)	C_6H_{14} (%)	$C_{12}N^a$ (%)
1	250	5	83	18	2	3	9	35	15	1
2	275	5	98	29	5	8	13	30	13	–
3	300	5	100	50	15	13	11	8	3	–
4	325	1	95	54	18	11	7	4	1	–
5	325	2	100	63	22	11	3	1	–	–
6	325	3	100	66	25	8	1	–	–	–
7	325	4	100	69	26	5	–	–	–	–
8	325	5	100	72	27	1	–	–	–	–
9	350	5	100	98	2	–	–	–	–	–

Reaction conditions: N-hexylhexanamide (250 mg), catalyst (25 mg, 0.5 wt% of Ru) and H_2 (50 bar). [a] $C_{12}N$ represents dihexylamine.

Supplementary Table 6 | Effect of temperature and reaction time on the product distribution of the hydrogenolysis of N-hexylhexanamide catalysed by Pt/CeO_2.

entry	T (°C)	time (h)	conversion (%)	CH_4 (%)	C_2H_6 (%)	C_3H_8 (%)	C_4H_{10} (%)	C_5H_{12} (%)	C_6H_{14} (%)	non-alkane products (%)
1	250	5	24	–	–	–	–	1	11	12
2	275	5	50	1	–	–	–	2	34	13
3	300	5	65	1	–	–	–	6	42	16
4	325	1	34	1	–	–	–	3	19	11
5	325	2	58	1	–	–	–	4	49	4
6	325	3	75	2	–	–	–	5	63	5
7	325	4	88	2	–	–	–	7	75	4
8	325	5	99	2	–	–	–	7	89	1
9	350	5	100	3	–	–	1	8	88	–

Reaction conditions: N-hexylhexanamide (250 mg), catalyst (25 mg, 0.5 wt% of Pt) and H_2 (50 bar). Detailed yields of non-alkane products are provided in Supplementary Table 8.

Supplementary Table 7 | Yields of non-alkane products from the hydrogenolysis of N-hexylhexanamide catalysed by Pt/CeO_2 under different temperatures and reaction times.

entry	T (°C)	time (h)	conv. (%)	alkane (%)	1 (%)	2 (%)	3 (%)	4 (%)
1	250	5	24	12	1	7	3	1
2	275	5	50	37	2	8	2	1
3	300	5	65	49	4	8	2	2
4	325	1	34	23	3	6	1	1
5	325	2	58	54	1	3	–	–
6	325	3	85	70	2	10	2	1
7	325	4	88	84	–	3	1	–
8	325	5	99	98	–	1	–	–

Chemical structures for **1**-**4** are provided in Supplementary Fig. 8.

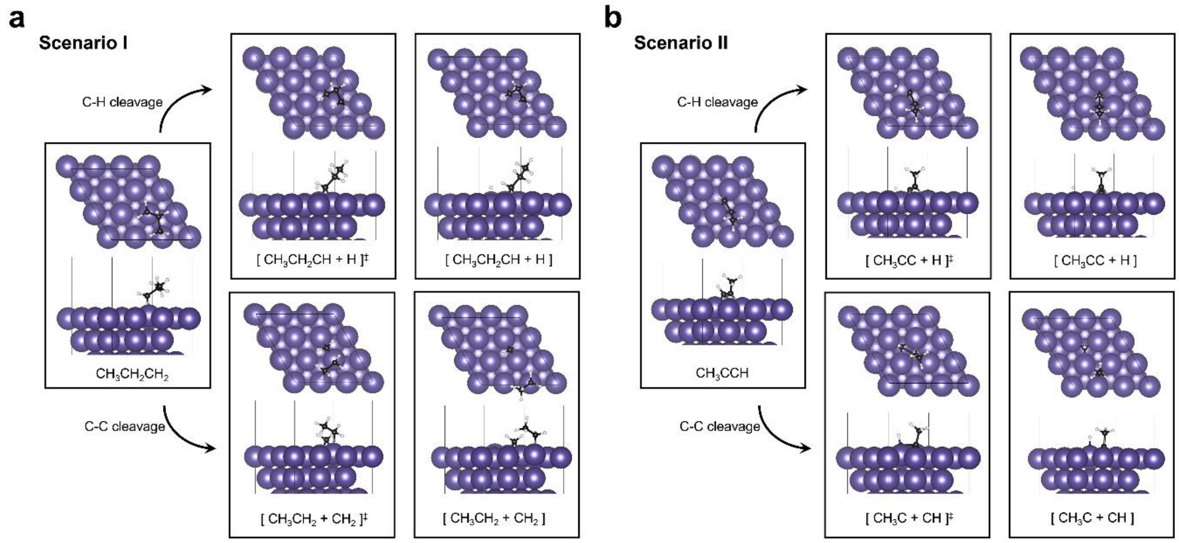

Supplementary Fig. 13 | DFT computations for hydrocarbon hydrogenolysis on Pt(111) surface. (a-b) Optimised geometries for C−H and C−C cleavage pathways in **a)** scenario I (high hydrogen-content C_3-species), and **b)** scenario II (low hydrogen-content C_3-species) on a Pt(111) surface.

Supplementary Table 8 | C−C and C−H bond cleavage pathways for scenario I and II.

Initial dehydrogenation:<br><br>$CH_3CH_2CH_3 \rightarrow [CH_3CH_2CH_2 + H] \rightarrow CH_3CH_2CH_2 + H$
Scenario I (high H content):<br><br>C−H bond cleavage:<br>$CH_3CH_2CH_2 \rightarrow [CH_3CH_2CH + H] \rightarrow CH_3CH_2CH + H$<br><br>C−C bond cleavage:<br>$CH_3CH_2CH_2 \rightarrow [CH_3CH_2 + CH_2] \rightarrow CH_3CH_2 + CH_2$
Scenario II (low H content):<br><br>C−H bond cleavage:<br>$CH_3CCH \rightarrow [CH_3CC + H] \rightarrow CH_3CC + H$<br><br>C−C bond cleavage:<br>$CH_3CCH \rightarrow [CH_3C + CH] \rightarrow CH_3C + CH$

Note: [ ] represents the as-dissociated species

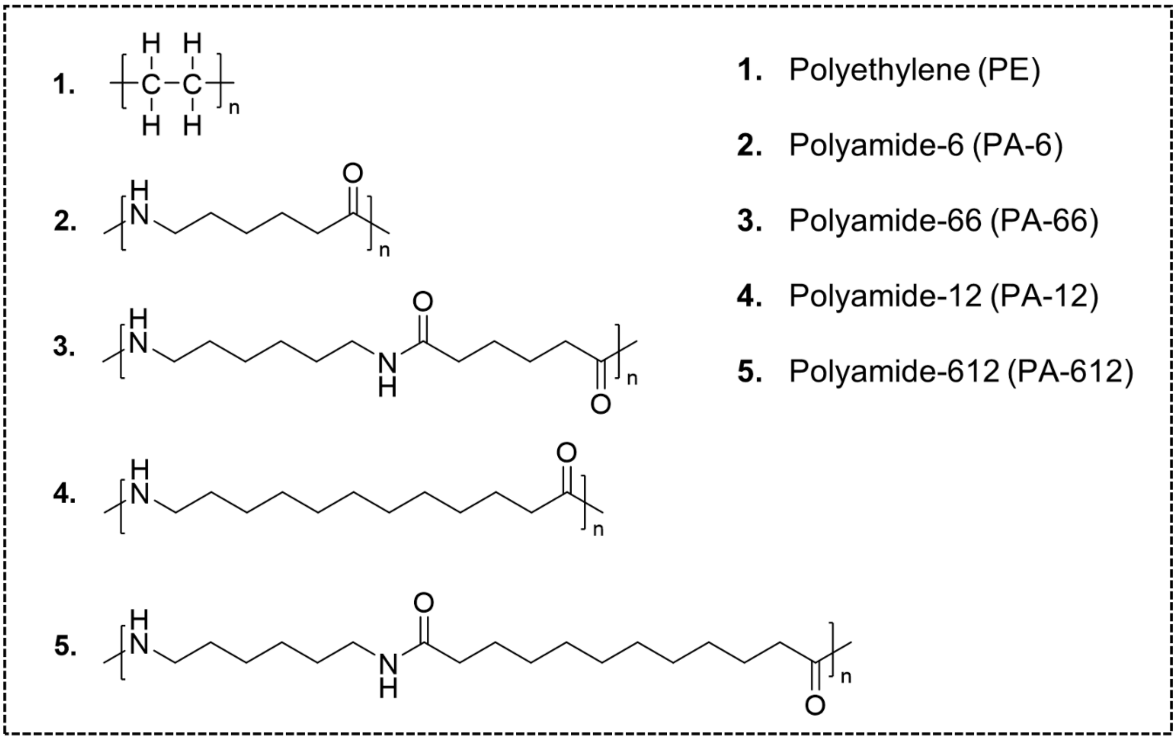

Supplementary Fig. 14 | Chemical structures of the polymers studied.

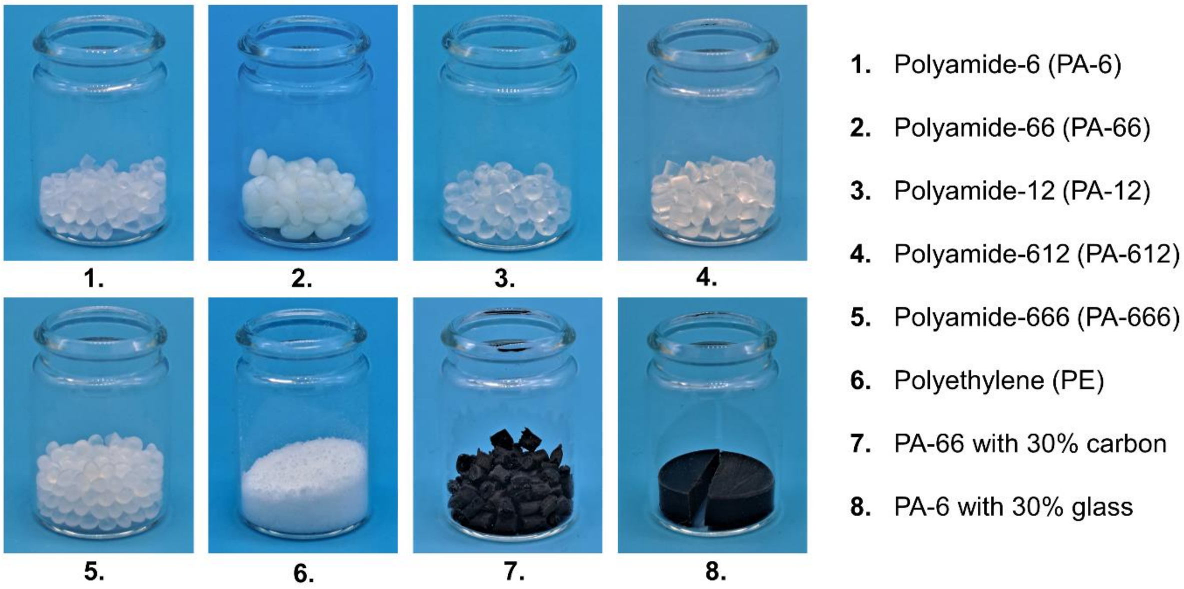

Supplementary Fig. 15 | Digital images of the polymer samples studied.

Supplementary Table 9 | Detailed conversions and product yields obtained from the hydrogenolysis of pure PAs, PA composites and PA blends.

entry	polymer	conversion (%)	CH_4 (%)	C_2H_6 (%)	C_3H_8 (%)	C_4H_{10} (%)	C_5H_{12} (%)	C_6H_{14} (%)	other alkanes[b] (%)	non-alkane products[b] (%)
**Ru/CeO_2**										
1	PA-6	100	96	4	–	–	–	–	–	–
2	PA-66	98	81	15	2	–	–	–	–	–
3	PA-12	100	99	1	–	–	–	–	–	–
4	PA-612	100	99	1	–	–	–	–	–	–
5	PA-66/carbon	99	76	8	4	4	2	1	2	2
6	PA-6/glass	99	61	7	7	9	7	3	2	3
7	PA-6/66	100	89	11	–	–	–	–	–	–
8	PA-6/PE	100	96	4	–	–	–	–	–	–
9	PA-6[a]	100	78	17	5	–	–	–	–	–
**Pt/CeO_2**										
10	PA-6	93	6	1	1	1	21	59	1	3
11	PA-66	85	4	1	1	1	20	49	4	5
12	PA-12	99	4	0	0	0	0	0	95	0
13	PA-612	96	6	1	1	1	8	29	48	2
14	PA-66/carbon	73	3	0	0	0	3	7	18	42
15	PA-6/glass	87	7	1	1	2	7	30	11	28
16	PA-6/66	77	6	0	0	1	18	46	2	4
17	PA-6/PE	43	3	0	0	1	7	27	4	1
18	PA-66/carbon[c]	88	8	0	0	3	13	40	8	16
19	PA-66/glass[c]	95	9	0	0	2	19	52	4	9

Reaction conditions: polymer (250 mg), catalyst (25 mg, 0.5 wt% of metal) and H_2 (50 bar) at 325°C for 24 h. [a] reaction was performed without stirring. [b] multiple species of amines, alcohols, and amides with varying degrees of isomerisation were produced, which were not quantified individually. [c] reaction was performed for 72 h. Supplementary Fig. 16 shows additional alkane products obtained from entries 12 and 13.

Supplementary Table. 10 | Additional alkane products and yields for the conversion of PA-12 and PA-612 with Pt/CeO_2.

polymer	products and yields
**PA-12**	*n*-$C_{10}H_{22}$ **2%**
	n-$C_{11}H_{24}$ **33%**
	n-$C_{12}H_{26}$ **60%**
**PA-612**	*n*-C_7H_{16} – *n*-$C_{10}H_{22}$ **6%**
	n-$C_{11}H_{24}$ **18%**
	n-$C_{12}H_{26}$ **30%**

Supplementary Table 11 | Product distribution obtained from the conversion of PA-6 at different reaction times.

entry	time (h)	conv. (%)	CH_4 (%)	C_2H_6 (%)	C_3H_8 (%)	C_4H_{10} (%)	C_5H_{12} (%)	C_6H_{14} (%)	others* (%)
**Ru/CeO_2**									
1	2	100	86	8	4	2	–	–	–
2	24	100	96	4	–	–	–	–	–
**Pt/CeO_2**									
3	12	65	2	–	–	1	11	34	17
4	18	86	4	–	–	1	15	64	2
5	24	93	6	1	1	1	21	59	4

Reaction conditions: PA-6 (250 mg), catalyst (25 mg, 0.5 wt% of metal) and H_2 (50 bar) at 325°C. *Includes other alkanes and non-alkane products.

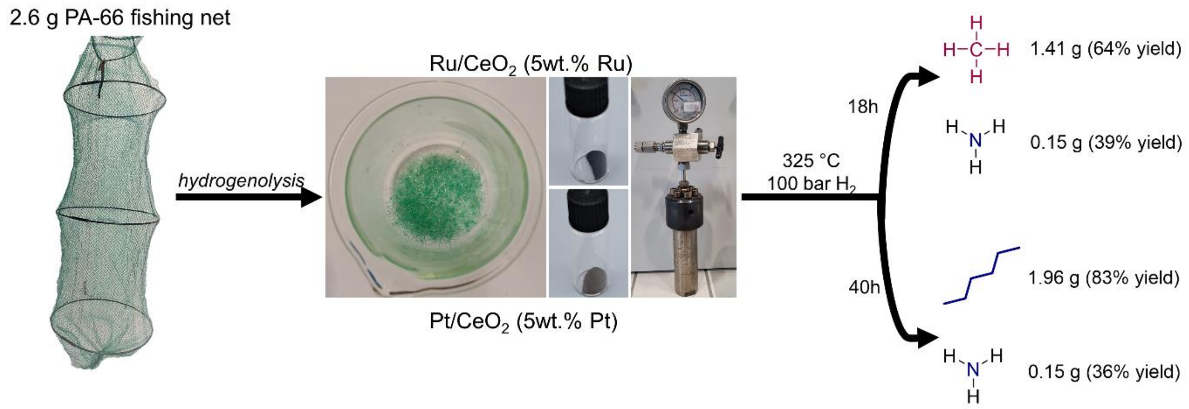

Supplementary Fig. 16 | Conversion of a PA-66 fishing net. Reaction conditions: PA-6 fishing net (2.6 g), catalyst (0.5 wt.% of metal) and H_2 (100 bar) at 325°C for 18 h (Ru/CeO_2) and 40 h (Pt/CeO_2).

Supplementary Table 12 | Recycling experiments of the Ru/CeO_2 catalyst evaluated via the hydrogenolysis of PA-6 for five reaction cycles

cycle	conv. (%)	CH_4 (%)	C_2H_6 (%)	C_3H_8 (%)	C_4H_{10} (%)	C_5H_{12} (%)	C_6H_{14} (%)
1	100	89.0	7.0	2.7	1.2	0.1	–
2	100	89.4	6.3	2.6	1.4	0.3	–
3	100	88.6	6.2	2.9	1.8	0.5	–
4	100	86.0	7.4	3.5	2.2	0.8	0.1
5	100	85.2	6.8	3.6	3.0	1.3	0.1

Reaction conditions: PA-6 (250 mg), catalyst (0.5 wt% of Ru) and H_2 (50 bar) at 325°C for 2 h.

Supplementary Table 13 | Recycling experiments of the Pt/CeO_2 catalyst evaluated via the hydrogenolysis of PA-12 for five reaction cycles.

cycle	conv. (%)	CH_4 (%)	$C_{10}H_{22}$ (%)	$C_{11}H_{24}$ (%)	$C_{12}H_{26}$ (%)
1	99.1	4.4	2.0	32.5	60.2
2	100	2.3	0.9	21.2	75.6
3	100	2.3	0.9	19.2	77.6
4	100	2.0	–	18.8	79.2
5	100	2.1	–	18.9	79.0

Reaction conditions: PA-12 (250 mg), catalyst (0.5 wt% of Pt) and H_2 (50 bar) at 325°C for 2 h.

Supplementary References

(1) Chaihad, N. et al. Catalytic pyrolysis of wasted fishing net over calcined scallop shells: Analytical Py-GC/MS study. *J. Anal. Appl. Pyrolysis* **146**, 104750 (2020).

(2) Wang, W., Meng, L., Huang, Y. Hydrolytic degradation of monomer casting nylon in subcritical water. *Polym. Degrad. Stab.* **110**, 312–317 (2014).

(3) Matsumoto, H., Akinari, Y., Kaiso, K., Kamimura, A. Efficient depolymerization and chemical conversion of polyamide 66 to 1,6-hexanediol. *J. Mater. Cycles Waste Manag.* **19**, 326–331 (2017).

(4) Czernik, S. et al. Catalytic pyrolysis of nylon-6 to recover caprolactam. *J. Anal. Appl. Pyrolysis* **46**, 51–64 (1998).

(5) Kamimura, A., Yamamoto, S. A novel depolymerization of nylons in ionic liquids. *Polym. Adv. Technol.* **19**, 1391–1395 (2008).

(6) Zhou, W. et al. Depolymerization of technical-grade polyamide 66 and polyurethane materials through hydrogenation. *ChemSusChem* **14**, 4176–4180 (2021).

(7) Coeck, R., De Bruyne, A., Borremans, T., Stuyck, W., De Vos, D. E. Ammonolytic hydrogenation of secondary amides: An efficient method for the recycling of long-chain polyamides. *ACS Sustain. Chem. Eng.* **10**, 3048–3056 (2022).
